# Study insights in the role of PGC-1α in neurological diseases: mechanisms and therapeutic potential

**DOI:** 10.3389/fnagi.2024.1454735

**Published:** 2025-02-12

**Authors:** Mi-bo Tang, Yi-xuan Liu, Zheng-wei Hu, Hai-yang Luo, Shuo Zhang, Chang-he Shi, Yu-ming Xu

**Affiliations:** ^1^Department of Geriatrics, The First Affiliated Hospital of Zhengzhou University, Zhengzhou University, Zhengzhou, China; ^2^Department of Neurology, The First Affiliated Hospital of Zhengzhou University, Zhengzhou University, Zhengzhou, China; ^3^NHC Key Laboratory of Prevention and Treatment of Cerebrovascular Diseases, Zhengzhou, Henan, China; ^4^Henan Key Laboratory of Cerebrovascular Diseases, Zhengzhou University, Zhengzhou, Henan, China

**Keywords:** PGC-1α, mitochondria, neuroinflammation, oxidative stress, neurological diseases

## Abstract

Peroxisome proliferator-activated receptor-*γ* coactivator-1α (PGC-1α), which is highly expressed in the central nervous system, is known to be involved in the regulation of mitochondrial biosynthesis, metabolic regulation, neuroinflammation, autophagy, and oxidative stress. This knowledge indicates a potential role of PGC-1α in a wide range of functions associated with neurological diseases. There is emerging evidence indicating a protective role of PGC-1α in the pathogenesis of several neurological diseases. As such, a deeper and broader understanding of PGC-1α and its role in neurological diseases is urgently needed. The present review provides a relatively complete overview of the current knowledge on PGC-1α, including its functions in different types of neurons, basic structural characteristics, and its interacting transcription factors. Furthermore, we present the role of PGC-1α in the pathogenesis of various neurological diseases, such as intracerebral hemorrhage, ischemic stroke, Alzheimer’s disease, Parkinson’s disease, Amyotrophic lateral sclerosis, Huntington’s disease, and other PolyQ diseases. Importantly, we discuss some compounds or drug-targeting strategies that have been studied to ameliorate the pathology of these neurological diseases and introduce the possible mechanistic pathways. Based on the available studies, we propose that targeting PGC-1α could serve as a promising novel therapeutic strategy for one or more neurological diseases.

## 1 Introduction

Mitochondria are vital organelles of eukaryotic cells that contain their own DNA.

In addition to ATP production, mitochondria play a crucial role in signaling and regulation across various pathways. Among these, calcium signaling stands out as a key mechanism through which mitochondria can regulate several cellular processes (Nunnari and Suomalainen, 2012). Mitochondrial biogenesis depends on the coordination between nuclear and mitochondrial DNA. The PPARγ coactivator-1 family (PGC-1) of transcription coactivators, including sirtuins and AMPK, is involved in regulating gene expression during mitochondrial biogenesis, as are mitochondrial transcription factor A (TFAM) and nuclear respiratory factors 1 and 2 (NRF1 and NRF2).

Neurons are high energy-demanding cells that require a tight regulation of Ca^2+^ to maintain their action potentials. Mitochondrial disorders have been reported in many neurological diseases. However, the exact mechanisms underlying the mitochondrial dysfunction vary between diseases. PGC-1α is conserved in eukaryotes and is particularly highly expressed in high-energy-consuming tissues and organs, such as brown adipose tissue, skeletal muscle, heart, liver, and brain. As a significant upstream molecule and downstream target of many vital pathways, PGC-1α is involved in the sophisticated crosstalk between neurons. In addition to acting as a master regulator of mitochondrial biogenesis (Jornayvaz and Shulman, 2010; Fan et al., 2024), PGC-1α also combats oxidative stress in neurons (Kong et al., 2010; Wang D. et al., 2022), protects against neuroinflammation (Ayasolla et al., 2005; Sun et al., 2024), functions as an upstream activator of neuro-autophagy (Tsunemi et al., 2012), and suppresses neuro-apoptosis (Wu et al., 2018). This review summarizes the current knowledge regarding the functions of PGC-1α in the brain and its role in neurological diseases, such as intracerebral hemorrhage (ICH), ischemic stroke, Alzheimer’s disease (AD), Parkinson’s disease (PD), Amyotrophic lateral sclerosis (ALS), and Polyglutamine (PolyQ) diseases. We hope that this review will facilitate future exploration of neurological diseases and promote the possible use of PGC-1α agonists/inhibitors as a new therapeutic strategy.

## 2 Role of PGC-1α in the central nervous system

**Figure 1:**
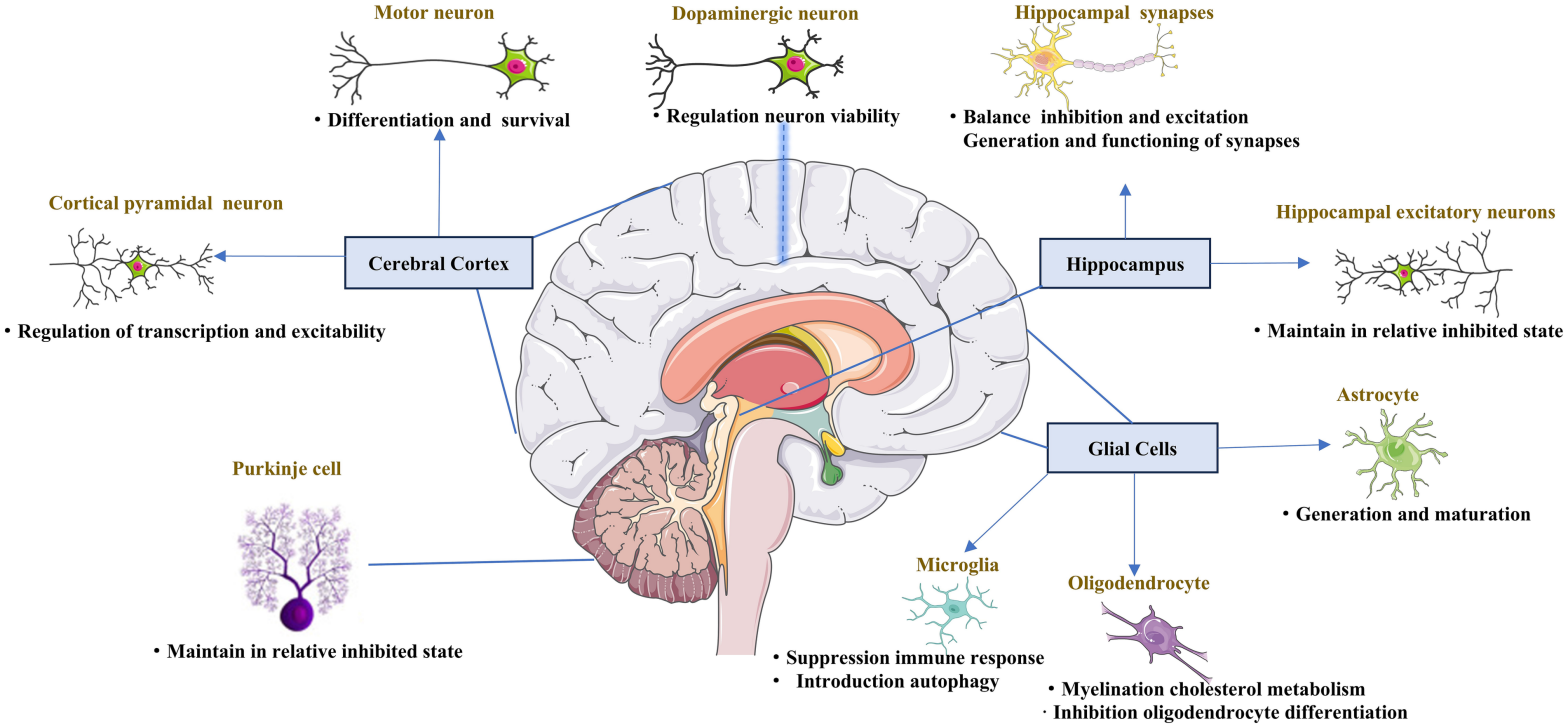
Role of PGC-1α in the central nervous system. The key role of PGC-1α in the maturation, differentiation, functioning, and survival of different neuron types across the central nervous system.

PGC-1α is abundant in the brain, particularly in the cerebral cortex, striatum, globus pallidus, and substantia nigra (SN), whereas it is absent in the hypothalamus (Tritos et al., 2003). The abnormal distribution of PGC-1α in the central nervous system, to some extent, explains its diverse roles in the physiological functions of different cell types (Figure 1).

### 2.1 Excitatory neurons of the cerebral cortex

PGC-1α expression is enriched in GABAergic interneurons expressing parvalbumin and glutamatergic projection neurons, particularly those located in the cortical pyramidal layer V, which contains both inhibitory and excitatory neurons (Cowell et al., 2007; Lucas et al., 2010). Both glutamatergic and fast-spiking interneuron populations depend on PGC-1α. In neocortical and hippocampal excitatory neurons, PGC-1α was shown to impair calcium homeostasis, increase ROS levels, and maintain cells in a relatively inhibited state to balance the inhibition and excitation, as the deletion of PGC-1α from neocortical and hippocampal excitatory neurons caused enhanced glutamatergic transmission in the neocortex and hippocampus (McMeekin et al., 2020). Motor neurons abundantly express PGC-1α. And increasing in PGC-1α mRNA are accompanied by voltage-gated currents characteristic of excitable cells and action potential formation when differentiating human embryonic stem cells or human neural stem cells into motor neurons (O'Brien et al., 2015). Moreover, the survival of motor neurons was also found to increase after treatment with lactate to induce CNS-specific PGC-1α (Bayer et al., 2017). Furthermore, the function of PGC-1α in motor neurons was also verified in studies of ALS (Bayer et al., 2017; Mehta et al., 2021). Together, these results indicate that PGC-1α is vital for the differentiation and function of motor neurons in the cerebral cortex.

### 2.2 Inhibitory neurons of the cerebral cortex and cerebellum

PGC-1α has heterogeneous functions in different nerve cells. Interneurons function to connect different types of CNS neurons, forming key nodes within brain neural circuitry. PGC-1α localizes in the nuclei of GABAergic interneurons, which form inhibitory synapses (Cowell et al., 2007). One study revealed that PGC-1α regulated inhibitory signaling via the control of the Ca^2+^–binding protein parvalbumin (Lucas et al., 2010), and the balance between inhibition and excitation of synapses in the hippocampus was also suggested to depend on sufficient levels of PGC-1α (Bartley et al., 2015; Wang et al., 2020). Moreover, PGC-1α upregulated the GABARα2 subunit in the hippocampus and frontal cortex, causing anxiety-like changes in the behavior of the mice (Vanaveski et al., 2021). Inhibitory signaling related to PGC-1*α* was also detected in Purkinje cells, the projection neurons in the cerebellum (Rosin et al., 2015). All these results demonstrate the essential function of PGC-1α in inhibitory signaling in the brain.

### 2.3 Midbrain dopaminergic neurons

Dopaminergic neurons are localized in the midbrain. PGC-1α is essential for the proper functioning of dopaminergic neurons, and is involved in the regulation of their viability. Researchers found that PGC-1α-knockout adult mice lost dopaminergic neurons in the substantia nigra (Jiang et al., 2016). Further, in the presence of overexpressed α-synuclein, dopaminergic neurons lacking PGC-1α were more predisposed to degradation (Ciron et al., 2015). A few studies further showed that growth factors interact with PGC-1α to regulate its function via mitochondrial biogenesis, oxidative stress, and neuroinflammation (Makela et al., 2014; Selvakumar et al., 2018; Fang et al., 2020). Several studies reported that PGC-1α was disadvantageous to dopaminergic neurons, as it might lead to developmental failures and the degeneration of dopaminergic neurons (Ciron et al., 2012; Clark et al., 2012). Together, the above information suggests that the levels of PGC-1α play a decisive role in whether it exerts protective or destructive effects.

### 2.4 Synaptogenesis

Synapses are vital for ensuring the nutritional requirements of neurons, particularly in mitochondrial transport. PGC-1α is also engaged in the process of synaptogenesis, with research showing that PGC-1α is involved in the development of dendritic spines and the formation of synaptic connections in the brain (Cheng et al., 2012). PGC-1α also influences the postnatal functioning of synapses, preserving hippocampal synapses, and promoting the postnatal generation of excitatory synapses of astrocytes (Cheng et al., 2012; Zehnder et al., 2021). Furthermore, PGC-1α regulates neurotransmitter release via control of the expression of the calcium sensors synaptotagmin 2 and complexin 1 (Lucas et al., 2014). Thus, PGC-1α is considered essential for the generation and functioning of synapses.

### 2.5 Spinal cord

Decrease in the level of PGC-1α was detected in regions affected by spinal cord injuries (SCI) compared to healthy tissue, suggesting that PGC-1α may be involved in the physiological functioning of the spinal cord (Hu et al., 2015). PGC-1α overexpression was found to decrease apoptosis in spinal cord neurons after SCI (Hu et al., 2016; Liu et al., 2017). Further studies in ALS mice showed that body-wide overexpression of PGC-1α decreased the degeneration of neuromuscular junctions (Liang et al., 2011). Activating PGC-1α ameliorated ischemic brain injury via PPARα-GOT1 axis by reducing the pyroptosis of endothelial cells, which was also reported (Wang et al., 2021). Microglial PGC-1α was found to be a promising therapeutic target for acute ischemic stroke by promoting autophagy and mitophagy through ULK1 and reducing NLRP3 activation (Han et al., 2021). Overall, these results suggest that PGC-1α may protect against spinal cord neuron failure, which is associated with the pathogenesis of many diseases, including ALS and stroke.

### 2.6 Glial cells of central nervous system

The most well-known function of glia in adults is controlling the formation of myelin sheaths around axons, which allows for the rapid conduction of signals essential for nervous system function (Jessen, 2004; Ludwig and Das, 2022). Glia also maintains appropriate concentrations of ions and neurotransmitters in the neuronal environment. There is an increasing body of evidence to indicate that glial cells also function as essential regulators of the formation, maintenance, and function of synapses, which are the key functional units of the nervous system (Hughes, 2021). The primary glial cell types in the CNS are astrocytes and oligodendrocytes. Astrocytes in the CNS regulate synapse formation and maintain the efficacy of synapses. PGC-1α was suggested to be important for the maturation of astrocytes (Zehnder et al., 2021). In neuronal models constructed from differentiated iPSCs, the activation of mitochondrial biogenesis was linked to upregulation of PGC-1α and the neuronal to glial fate decision switch (Augustyniak et al., 2019). Research further showed that deletion of PGC-1α resulted in the extended proliferation and hindered astrocyte morphogenesis (Zehnder et al., 2021). The central function of oligodendrocytes is to generate myelin, which is an extended membrane from the cell that wraps tightly around axons. Indeed, research reported that there is a considerable increase in PGC-1α during myelination in rat brain (Cowell et al., 2007). PGC-1α also plays several roles in myelination by regulating MBP expression and cholesterol metabolism in oligodendrocytes (Xiang et al., 2011). Activation of PGC-1α by exercise has been shown to be linked to the enhancement of myelin thickness (Jensen et al., 2018). In one *in vitro* study, PGC-1α was shown to be involved in the protection of oligodendrocyte progenitor cells (OPCs) under inflammatory conditions.

## 3 Structure of PGC-1α

**Figure 2:**
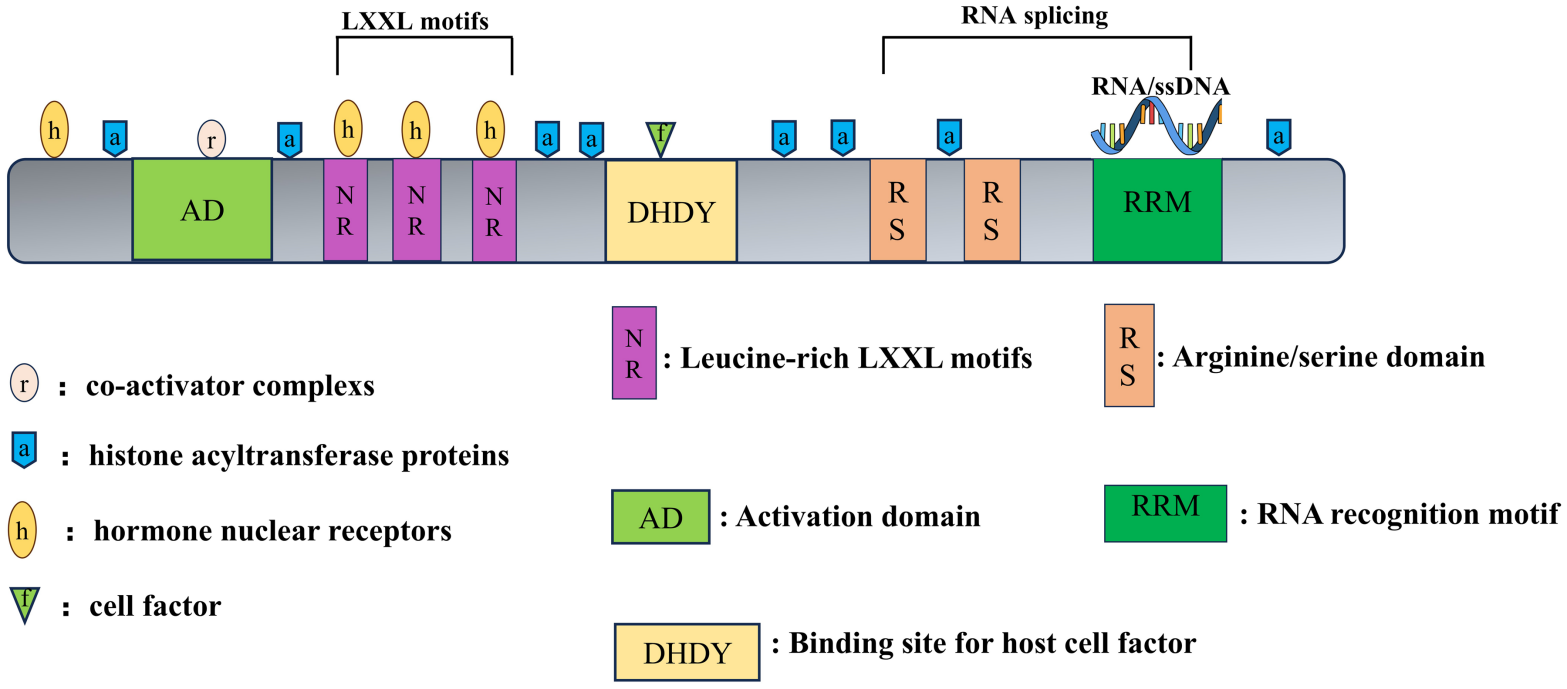
Structure of PGC-1α. The amino-terminal region of PGC-1α functions as the surface for the recruitment of histone acyltransferase proteins. This domain contains several LXXL motifs (NR boxes), acting as coactivator signature essential for interactions with multiple types of other transcription factors and nuclear receptors. The carboxy-terminal contains RRM, which act as binding site of RNA and single-stranded DNA. The binding site for host cell factor, DHDY, synergizes with HCF to regulate the cell cycle and enhance the transcriptional activity of PGC-1α. Another RS domain plays an important role in mRNA splicing.

As a transcriptional coactivator, PGC-1α contains several domains with various functions. PGC-1 coactivators exert their transcriptional regulatory function by binding to a variety of transcription factors and nuclear receptors that recognize specific sequences on target genes (Villena, 2015). The amino-terminal region of PGC-1α contains a highly conserved activation domain that serves as a surface for the recruitment of histone acetyltransferase proteins. This domain also contains several leucine-rich LXXL motifs, also termed NR boxes, which drive a coactivator signature essential for interactions with multiple types of other transcription factors and nuclear receptors (Monsalve et al., 2000). The carboxy-terminal contains a well-conserved RNA recognition motif (RRM), which is involved in the binding of both RNA and single-stranded DNA. Short serine/arginine-rich stretches, called RS domains, are also located at the N-terminal of the RRM motif in PGC-1α (Monsalve et al., 2000), and they play an important role in mRNA splicing, although their role in disease is unclear. The C-terminal region of PGC-1α also has a binding site for host cell factor (HCF), also known as DHDY, which synergizes with HCF to regulate the cell cycle and enhance the transcriptional activity of PGC-1α (Lin et al., 2002). Finally, several binding sites for other coregulators, including MEF-2C, yin yang-1 (YY1), PPARγ, forkhead box O1 (FoxO1), mediator complex subunit 1 (MED1), and BRG1-associated factor 60a, are also located in the C-terminal region of PGC-1α (Villena, 2015; Ma et al., 2018; Xin et al., 2018). The intermediate connector is a multiunit complex that is required for transcription (Figure 2).

## 4 Research progress regarding PGC-1α in neurological diseases

**Figure 3:**
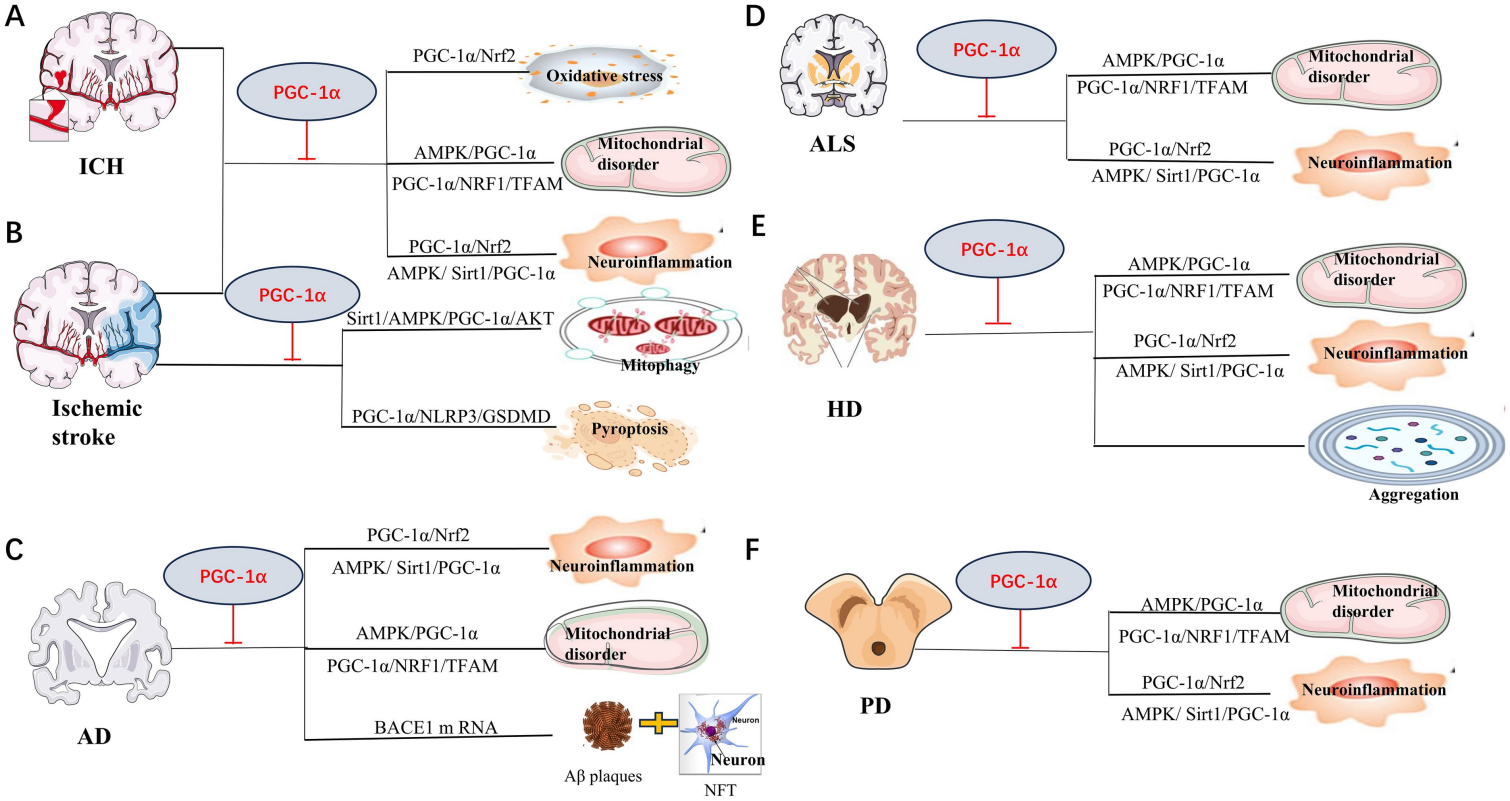
Mechanisms of PGC-1α involved in neurological diseases. Mitochondrial disorder, neuroinflammation and oxidative stress are the main pathway for PGC-1α amelioration of neurological disorders in Intracerebral hemorrhage (ICH) **(A)**, ischemic stroke **(B)**, Alzheimer’s disease (AD) **(C)**, Huntington disease (HD) **(D)**, Amyotrophic lateral sclerosis (ALS) **(E)** and Parkinson’s disease (PD) **(F)**. Repair of mitochondria mediated by PGC-1α plays a crucial role in the protection against ICH injury. Recent study also demonstrated that pyroptosis was another vital process for ischemic stroke. Study suggest that PGC-1α preventing ischemic stroke via reducing pyroptosis. PGC-1α could also suppress BACE1 mRNA, which is important for generation of Aβ, to protect AD. In HD, PGC-1α was also found to decrease aberrant aggregation and turnover of mutant HTT. The specific pathways as shown.

Neurons are high-energy-demanding cells, whereas PGC-1α is abundantly expressed in the CNS. Many studies of the role of PGC-1α in neurological diseases, such as ICH, ischemic stroke, AD, PD, ALS and PolyQ diseases, have been conducted. The main pathogenesis of these disorders included mitochondrial dysfunction, oxidative stress, neuroinflammation, autophagy, etc. (Figure 3). Therefore, we review the main roles and pathways of PGC-1α in neurological diseases below.

### 4.1 Intracerebral hemorrhage

Intracerebral hemorrhage (ICH) is a life-threatening disease of global importance, with poor prognosis and few efficient treatment options (Cordonnier et al., 2018). Previous reports indicate that the mortality rate among ICH patients is approximately 40% within one month (van Asch et al., 2010). ICH accounts for 10–30% of all strokes, affecting more than one million people every year worldwide (Feigin et al., 2003). The pathology of ICH involves mechanical disruption following initial bleeding and oxidative stress, inflammation, mitochondrial dysfunction, and neuronal death (Aronowski and Zhao, 2011; Yu et al., 2019). Abundant evidence supports the hypothesis that mitochondrial dysfunction occurs after ICH (Wu et al., 2020; Li et al., 2021), which is reportedly linked with both structural and functional disorders (Zhou et al., 2017; Xu et al., 2019). By injecting autologous arterial blood into rat brains, researchers found that the level of PGC-1α increases significantly at 1 h after ICH injury, reaching a peak at 72 h (You et al., 2016). Studies in an ICH rat model showed that treatment with PGC-1*α* siRNAs significantly decreased ATP concentration, the number of mitochondria, mitochondrial proteins, and mitochondrial DNA, increased brain water content, and led to the formation of mitochondrial myelin layer structures (Zhou et al., 2017). The activation or overexpression of PGC-1α after ICH injury promoted mitochondrial biogenesis and mitochondrial-related ROS metabolism, reduced mitochondrial dysfunction, inhibited mitochondrial-dependent cell death, and prevented the further occurrence of secondary brain injury (You et al., 2016; Zhou et al., 2017; Jin et al., 2022). Further studies showed that PGC-1*α* alleviated mitochondrial dysfunction and secondary brain injury following ICH via AMPK- PGC-1α pathway (Yu et al., 2019). Studies in recent years also demonstrated that PGC-1α involvement in mitochondrial biogenesis and antioxidative capability is protective for mice following ICH (Zhang et al., 2024).

### 4.2 Ischemic stroke

More than 80% of strokes are ischemic and caused by an occlusion of cerebral arteries (Lapchak and Zhang, 2017). Lack of blood supply results in neurons being deprived of necessary glucose and oxygen (OGD), which finally triggers pathophysiological processes including excitotoxicity, oxidative stress, inflammation, apoptosis, and cell death. Though the introduction of tissue plasminogen activator for the acute treatment of ischemic was achieved (Yoshimura et al., 2018), development of neuroprotective agents for treating ischemia outside of the current narrow therapeutic window is still much more vital. There is significant experimental evidence supporting the roles of mitochondrial dysfunction and oxidative stress as determinants of neuronal death, as well as endogenous protective mechanisms after stroke (St-Pierre et al., 2006; Yin et al., 2008; Chen et al., 2010). Several studies reveal that PGC1-α contributes to neuronal survival (St-Pierre et al., 2006; Chen et al., 2011). ROS plays a crucial role in the fate of neurons, as well as in damage progression following ischemic stroke (Mattiasson et al., 2003). PGC-1α is a master regulator of the mitochondrial proteins SOD2 and UCP2. UCP2 and SOD2 are vital ROS-detoxifying proteins found in mitochondria. Studies revealed that the upregulation of UCP2 decreases ROS and neuronal loss in the brain tissue after cerebral ischemia (Mattiasson et al., 2003; Chen et al., 2006; Deierborg et al., 2008). All the above information demonstrates that PGC1-α protects neurons from ischemic stroke by improving mitochondrial function. Advances in neuroscience have prompted further exploration of the mechanisms by which PGC-1α provides protection. Pyroptosis, a type of programmed cell death has been demonstrated to be involved in ischemic stroke in recent years (Zhang et al., 2019). And, study found that medioresinol, a PGC-1α activator, ameliorated the pyroptosis of endothelial cells and ischemic brain injury via PPARα-GOT1 axis (Wang et al., 2021). Neferine, a bisbenzylisoquinoline alkaloids extracted from Plumula Nelumbinis.

was also found to benefit ischemia/reperfusion injury by inhibiting pyroptosis, reducing mitochondrial oxidative stress, ameliorates endothelial inflammation via regulation of PGC-1α/NLRP3/GSDMD signaling pathway (Zheng et al., 2024). Mitophagy, which is beneficial in ischemic stroke, was also triggered by USP18/FTO via the SIRT6/AMPK/PGC-1α/AKT pathway in recent studies (Song et al., 2024). Studies have revealed that neuroinflammation and immune responses occur minutes to hours after stroke (Fu et al., 2015). PGC-1α in microglia could also protect against the ischemic brain injury by suppressing neuroinflammation (Wang et al., 2018; Han et al., 2021). Further, changes in the level of PGC-1α after stroke are reported for decades (Valerio et al., 2011; Wang et al., 2014). Therefore, targeting PGC-1α is a promising therapeutic strategy for ischemic stroke. Compounds and drugs targeting PGC-1α that have been explored for improving ischemic stroke in past decades are also collected (Table 1).

**Table 1:** Compounds and drugs which have been found to improve ischemic stroke in past decade studies.

Compound/Drug	Possible pathway	Reference
Medioresinol	Activating PPARα-GOT1 axis	Wang et al. (2021)
Mangiferin	Activating SIRT1/PGC-1α	Chen et al. (2021)
Alpha-lipoic acid nitrone	Up-regulating SIRT1- PGC-1α	Fu et al. (2015)
Dl-3n-butylphthalide	Regulating eNOS-PGC-1α of endothelial	Wei et al. (2019)
Icariin	Increasing SIRT1/PGC-1α	Zhu et al. (2010)
Cerebrolysin	Activating CREB/PGC-1α	Guan et al. (2019)
Donepezil	Activating PGC-1α	Madani et al. (2021)
Naoxinqing	Activating AMPKα/NAMPT/SIRT1/PGC-1α	Sun et al. (2024)
3C	Activating CaMKKβ/AMPK/PGC-1α	Wang et al. (2018)
Ubiquitin-specific peptidase 18	Activating SIRT6/AMPK/PGC-1α/AKT	Song et al. (2024)

### 4.3 Alzheimer’s disease

Close to 50 million people worldwide are currently suffering from dementia, including AD, whose prevalence is estimated to surpass 100 million by 2050. AD, a progressive neurodegenerative disease, is the most common form of dementia (Blennow et al., 2006; Alzheimer's Association, 2016). Age, family history, apolipoprotein E ɛ4 genotype, hypertension, obesity, diabetes, traumatic brain injury, hyperlipidemia, and low education level are all considered risk factors for AD (Blennow et al., 2006; Alzheimer's Association, 2016). Synaptic dysfunction, neurotransmitter imbalance, neuroinflammation, infection, gut microbiome disruption, genetic mutations, oxidative stress, and autophagy are all potential pathogenic factors influencing AD (Crews and Masliah, 2010; Barage and Sonawane, 2015; Ferreira-Vieira et al., 2016; De-Paula et al., 2018; Giau et al., 2018; Ashraf et al., 2019; Khan et al., 2020). Aggregation of A*β* peptides in neuritic plaques and neurofibrillary tangles (NFTs) of hyperphosphorylated Tau protein are pathological features of AD (Amemori et al., 2015). Generation of Aβ depends on the sequential proteolytic cleavage of the amyloid precursor protein (APP) by β-secretase 1 (BACE1) and the *γ*-secretase complex (Hardy and Selkoe, 2002). APP/PS1 mutated mice are classical models for AD investigation, with research showing that enhanced PGC-1*α* mRNA levels caused by decreased function of PS1 may be involved in the pathogenesis of this research model (Robinson et al., 2014). Given that BACE1 is the rate-limiting enzyme in Aβ generation and APP processing, it is suggested to be one of the most important therapeutic targets for treating AD. PGC-1α is shown to decrease in the brains of AD patients (Zehnder et al., 2021). Studies revealed that overexpression of PGC-1α suppressed the basal transcription of endogenous BACE1 mRNA (Wang et al., 2013),which further explains the phenomenon that levels of PGC-1α protein are inversely linked to both AD-type neuritic plaque abnormalities and Aβ content (Pohland et al., 2018). Further research showed that the effect of PGC-1 on BACE1 requires deacetylation by SIRT1 (Wang et al., 2013), and it has also been demonstrated that metabolic stress modulates Alzheimer’s β-secretase gene transcription via action on the SIRT1-PPAR*γ*-PGC-1 pathway. Mitochondrial dysfunction is a pivotal event in AD pathogenesis (Panes et al., 2020). Repairment of mitophagy and mitochondrial biogenesis ameliorated AD via PINK1/LC3B/P62 and PGC-1*α*/Nrf2 in one study of Lithospermic acid B (Meng et al., 2024). SIRT1/PGC-1α signaling pathway, which reduces oxidative stress, was also found to attenuate cognitive deficits in Alzheimer’s disease (Liu et al., 2023). Neuroinflammation is also known to influence Aβ plaques and NFT. Indeed, many studies support the idea that Aβ plaques and neuroinflammation form a positive feedback loop in AD (Doig, 2018). One study found that resveratrol (RSV) reduced inflammatory cytokine release, improved mitochondrial bioenergetics, and ameliorated Aβ-peptide clearance by activating SIRT1 and AMPK (Price et al., 2012). The latter functions in the AMPK/SIRT1/PGC-1α pathway, a canonical signaling cascade influencing cell survival (Tian et al., 2019; Zhang and Wu, 2021). PGC-1α as the media of regulation mitochondrial biogenesis, mitophagy, neuroinflammation and Aβ generation involved in the pathogenesis and neuroprotection of AD.

### 4.4 Parkinson’s disease

PD is one of the most common neurodegenerative disorders worldwide, and the number of people suffering from this disorder has more than doubled in the last 30 years. The pathological features of PD include the progressive loss of dopaminergic neurons (Meara, 1994). The possible pathophysiologic mechanisms underlying PD include α-synuclein misfolding and aggregation, mitochondrial dysfunction, impairment of protein clearance, neuroinflammation, and oxidative stress (Jankovic and Tan, 2020). The involvement of mitochondrial disorders in PD was first established more than three decades ago. In 1983, researchers found that the administration of 1-methyl-4-phenyl-1,2,3,4-tetrahydropyridine (MPTP) led to parkinsonism in both animal models and humans through metabolite ion MPP+ activation and the subsequent inhibition of multiple complexes of the respiratory chain (Langston and Ballard, 1983; Schapira et al., 1989; Grunewald et al., 2019). Subsequent research achieved significant progress regarding the elucidation of the monogenic causes of PD associated with mitochondrial dysfunction. These genes include SNCA (Polymeropoulos et al., 1997), LRRK2 (Paisan-Ruiz et al., 2004), VPS35 (Tang et al., 2015), CHCHD2 (Funayama et al., 2015), Parkin, PINK1, and DJ-1. Another study of MPTP found that LSN862 (LSN), a novel non-thiazolidinedione partial PPAR- γ agonist, exerted neuroprotective effects in a mouse model of PD by increasing PPAR- γ and PGC1-α expression (Swanson et al., 2013). AMPK/SIRT-1/PGC-1α-mediated autophagy (Lee et al., 2023; Elesawy et al., 2024), mitochondrial biogenesis (Liu et al., 2022; Jhuo et al., 2024) PGC-1α/Nrf mediated oxidative stress (Guo et al., 2023) were all classical pathway in PD. Research further identified many molecules and drugs that can ameliorate PD (Table 2). For example, findings in a 6-hydroxydopamine (6-OHDA)-lesioned rat model of PD revealed that ferulic acid reduced mitochondrial Drp1 and increased PGC1α, restoring mitochondrial dynamics in lesioned animals (Anis et al., 2020). PGC-1α also protects neurons in PD from neuroinflammation (Mohammed et al., 2024). Previous work also found that *L. plantarum* DP189 activated the (Nrf2)/ARE and PGC-1α pathways and suppressed the NLRP3 inflammasome in PD mice (Wang L. et al., 2022). To date, the study of mitochondria dysfunction and the methods to reverse this disorder play major role in the field of PD research. Moreover, additional studies on other physiological processes of PD are also needed.

**Table 2:** Compounds and drugs which have been found to improve PD in past decade studies.

Compound/Drug	Possible pathway	Reference
Empagliflozin	Activating AMPK/SIRT-1/PGC-1 and wnt/β-catenin	Mohammed et al. (2024)
Farnesol	Enhancing farnesylation of PARIS and restoring PGC-1α activity	Jo et al. (2021)
Tetramethylpyrazine nitrone	Activating PGC-1α/Nrf2	Guo et al. (2023)
liraglutide	Upregulating PGC-1α	Wu et al. (2022)
Roflupram	Activating CREB/PGC-1α	Zhong et al. (2020)
6-Hydroxydopamine	Activating PKA/Akt/GSK-3β and CREB/PGC-1α/NRF-1/TFAM	Zhou et al. (2019)
Urolithin A	Activating SIRT1-PGC-1α	Liu et al. (2022)
Alpha-lipoic acid (ALA)	Upregulating Sirt1/PGC-1α	Zhang et al. (2022)
Teaghrelin	Activating AMPK/SIRT1/PGC-1α and ERK1/2	Jhuo et al. (2024)
Fucoidan	Activating AMPK-PGC-1α	Han et al. (2019)
Garcinol	DJ-1/SIRT1/PGC-1/ p-AMPK	Lee et al. (2023)

### 4.5 Amyotrophic lateral sclerosis

ALS is a fatal neurodegenerative disease. As a rare and poorly characterized disorder, it is difficult to diagnose. The lifetime risk of ALS is high, with approximately 1 in 350 people ultimately developing the disease, although limited life expectancy reduces the prevalence (Ryan et al., 2019). As a combination disorder of upper and lower motor neurons, ALS presents as progressive weakness of the voluntary skeletal muscles involved in limb movement, swallowing (dysphagia), speaking (dysarthria), and respiratory function, with heterogeneous clinical presentations. Unlike other neurodegenerative diseases, the pathogenesis of ALS remains ambiguous, and the pathogenic genes are complex. Although the frequency of genetic subtypes does vary by race, the most common mutations are found in C9orf72, TARDBP, SOD1, and FUS (Smith et al., 2013). Studies support the hypothesis that impaired RNA metabolism, altered proteostasis, autophagy, trafficking defects, and mitochondrial dysfunction are the primary pathological processes underlying ALS (Nguyen et al., 2018). As an indispensable molecule for cells, PGC-1α is decreased in the CNS due to mutations in SOD1 and FUS/TLS in two mouse models of familial ALS (Thau et al., 2012; Bayer et al., 2017). Lactate-induced CNS-specific PGC-1α pathway activation was also completely absent in ALS patient-derived motoneurons with two different frame-shift FUS/TLS mutations (Bayer et al., 2017). PGC-1α was also considered a male-dominant disease modifier of ALS (Eschbach et al., 2013). Increasing mitochondrial mass through PGC-1α over-expression led to protection against ALS neurodegeneration as shown (Varghese et al., 2020). Research in the SOD1^G93A^ ALS mouse model showed that activation of PGC-1α/Nrf2/HO-1 pathway increased mitochondrial antioxidant activity and decreased expression of human SOD1 (Wen et al., 2021), one pathogenic protein of ALS. AMPK/PGC-1α/Nrf1/Tfam activation promotes mitochondrial biogenesis and ameliorates mitochondrial dysfunction, which was also verified in SOD1^G93A^ mice. Indeed, numerous studies have identified several molecules/drugs which can treat ALS by modulating the PGC-1α pathway (Table 3), which offers a promising prospect for targeting PGC-1α as effective treatment strategies.

**Table 3:** Compounds and drugs which have been found to improve ALS in past decade studies.

Compound/Drug	Possible pathway	Reference
R13, a prodrug of 7,8-dihydroxyflavone	Activating AMPK/PGC-1α/Nrf1/Tfam	Li et al. (2021)
Tetramethylpyrazine nitrone	Activating PGC-1α/Nrf2/HO-1	Wen et al. (2021)
Tetramethylpyrazine nitrone	Activating Akt/mTOR/GSK-3β and AMPK/PGC-1α/Nrf2	Huang et al. (2021)
VAR10303	Upregulating PGC-1α	Golko-Perez et al. (2017)
Oxaloacetate	Increase TNFα andPGC-1α mRNAs	Tungtur et al. (2021)

### 4.6 Huntington’s disease

HD is an autosomal dominant neurodegenerative disease caused by the expansion of CAG repeats in the first exon of the HTT gene, encoding a mutant Huntington (mHTT) protein (Leavitt et al., 2020). HD is characterized by atrophy of the basal ganglia nuclei and subcortical white matter, and its associated symptoms include motor, psychiatric, and cognitive deficits, which generally present in middle age and are gradually progressive (Tabrizi et al., 2019). There is still no effective treatment for HD. A toxic gain-of-function of the mHTT protein, caused by expanded PolyQ, has been proposed as a driving factor in HD, which functions by leading to neuronal loss. The mechanisms underlying the disease are complex, including deregulation of the ubiquitin, proteasome, and autophagy systems, as well as oxidative stress (Jimenez-Sanchez et al., 2017). Recently, mitochondrial dysfunction was identified as a key contributor to HD pathology (Kumar and Ratan, 2016; van Well et al., 2019; Jurcau and Jurcau, 2023). Studies in HD animal models and the striatal tissues of patients have shown that transcriptional dysregulation of PGC-1α is involved in the neurodegeneration of HD (Weydt et al., 2009; La Spada, 2012). Transcription and mitochondrial dysfunction caused by alteration in PGC-1α were also documented in HD (Weydt et al., 2006). Though most studies support that energy failure is involved in the role of PGC-1α disorder in HD, disruption of ribosomal transcription by PGC-1α was also identified in HD (Jesse et al., 2017). On the other hand, the role of CREB in the regulation of PGC-1α in neurons in response to oxidative stress was studied (St-Pierre et al., 2006). PGC-1α promoter binding with less HSF1 in a cell culture model of HD demonstrated the destruction of the heat shock response in HD (Intihar et al., 2019). Less activation of HSF1 in this model exacerbates cell death (Intihar et al., 2019). The role of PGC-1α in the pathogenesis of HD was further verified by PGC-1α KO models emulating the main characteristics of HD (Lin et al., 2004; Jesse et al., 2017). Crossing N171-82Q HD transgenic mice with inducible TRE-PGC-1α mice decreased aberrant aggregation and turnover of mutant HTT, mitigated striatal neurodegeneration, and improved mitochondrial dysfunction observed in HD mice (Tsunemi et al., 2012). Several drugs and compounds ameliorating the phenotype of HD via regulating PGC-1α have also been identified in recent decades (Table 4).

**Table 4:** Compounds and drugs which have been found to improve HD in past decade studies.

Compound/Drug	Possible pathway	Reference
Morin hydrate	Enhancing p-PGC-1α and p-VDAC1	El-Emam et al. (2024)
ginsenoside Rg3 and Rf	Increase PGC-1α and p-CREB	Lee et al. (2021)
Beta-Lapachone	Activating Sirt1/p-CREB/ PGC-1α	Lee et al. (2018)
Resveratrol	Activating PGC-1α/TFAM	Naia et al. (2017)
Epoxyeicosatrienoic acid	Increase PGC-1α	Waldman et al. (2016)
Metformin	Increase PGC-1α	Di Cristo et al. (2019)
Bezafibrate	Increase PGC-1α	Chandra et al. (2016)
Teaghrelin	Activating AMPK/SIRT1/PGC-1α and ERK1/2	Jhuo et al. (2024)

### 4.7 Other PolyQ diseases

Spinocerebellar ataxia 3 (SCA3), which is one of the nine identified PolyQ diseases, is a rare, inherited neurodegenerative disease. As the second most prevalent disease in the PolyQ group, SCA3 affects about 1–5 in every 100,000 people globally (Li et al., 2018). Mitochondrial dysfunction, metabolic abnormalities, autophagy and oxidative stress have been shown to be involved in the pathogenesis of SCA3. As a vital molecule controlling mitochondrial biogenesis, PGC-1α has also been demonstrated to ameliorate SCA3. Glycyrrhiza inflata herb extract and its constituents, licochalcone A and ammonium glycyrrhizinate, increased mitochondrial biogenesis, decreased oxidative stress, and reduced aggregate formation in SCA3 cellular models via the activation of PGC-1α and NRF2-ARE (Chen et al., 2014). Further, SG-Tang has been proven to exert protective effects against SCA17, another PolyQ disease characterized by progressive ataxia, seizures, cognitive dysfunction, psychiatric symptoms, and pyramidal/extrapyramidal signs via upregulating the PGC-1α/SOD2/CYCS, NRF2/GCLC/NQO1, and NFYA/HSPA5 pathways (Chen et al., 2019). Although studies of PGC-1α in other PolyQ diseases are limited, research in this field is ongoing and has yet to be fully explored.

## 5 Conclusion

Neurological diseases are the leading cause of disability and the second leading cause of death worldwide. With population aging, the number of patients suffering from neurological disease is rising. While stroke remains the most common neurological disease, morbidity related to neurodegenerative diseases, such as AD and PD, is becoming increasingly common. The incidences of hereditary neurological diseases, like HD, SCA (SCA1, SCA2, SCA3, SCA7, SCA36, etc) are also increasing year by year. Reducing morbidity and increasing effective therapeutic strategies for neurological disorders are therefore urgently needed.

In this review, we highlight the role of PGC-1α in the nervous system and neurological diseases such as stroke, AD, PD, ALS, and PolyQ diseases. A review of the existing literature suggests that PGC-1α overexpression could be a potential therapeutic target for these types of diseases. However, almost all studies upregulated PGC-1α via gene transfection and compound drugs *in vitro* or *in vivo* animal models. Whether PGC-1α supplementation and which compounds could open promising avenues for neuroprotective therapeutics among neurological diseases, or even within a specific neurological disease, merits further rigorous preclinical studies and decisive evaluation through clinical trials.
